# Cryopreserved Thyroid Tissue Autotransplant in Pediatric Age Patients: A Feasibility Study and Literature Review

**DOI:** 10.3390/cancers16112112

**Published:** 2024-05-31

**Authors:** Claudio Spinelli, Marco Ghionzoli, Linda Idrissi Sahli, Silvia Visintainer, Carla Guglielmo, Chiara Cordola, Simone Lapi, Elisa Biagi, Angela Pucci, Riccardo Morganti, Silvia Martina Ferrari, Alessandro Antonelli

**Affiliations:** 1 Pediatric Surgery Unit, Maternity and Children Department, University of Pisa, 56124 Pisa, Italy; 2 Biobank Division, University Hospital of Pisa, 56124 Pisa, Italy; 3 Department of Surgical, Medical and Molecular Pathology and Critical Area, University of Pisa, 56124 Pisa, Italy; 4 Section of Statistics, University Hospital of Pisa, 56124 Pisa, Italy; 5 Department of Clinical and Experimental Medicine, University of Pisa, 56124 Pisa, Italy

**Keywords:** thyroid, children, autotransplant, cryopreservation

## Abstract

**Simple Summary:**

This study explores cryopreservation thyroid autotransplantation as an alternative to hormonal replacement therapy for patients who underwent thyroidectomy during childhood or adulthood. The research evaluates the impact of modern cryopreservation techniques on follicular cell integrity through a feasibility study and literature review. Results indicate preserved tissue architecture and cell viability. Animal and human studies demonstrate successful transplantation outcomes. However, long-term follow-up data are lacking. The findings suggest promising viability of cryopreserved thyroid tissue, yet further research is needed to assess hormone secretion and ensure sustained functionality. This alternative therapy could offer tailored hormone production, aligning with physiological needs.

**Abstract:**

Background and aims: This paper aims to study an alternative solution to hormonal replacement therapy in specific groups of patients who underwent thyroidectomy during childhood or adulthood. After cryopreservation, thyroid autotransplantation could be an alternative solution which would allow us to use the ability of the thyroid tissue of producing hormones according to the physiological needs of the body. Materials and methods: A feasibility study about the effects of the most modern cryopreservation techniques on the structural and functional integrity of the follicular cells of the thyroid tissue has been carried out. Patients who could benefit from the treatment have been found for both autotransplant techniques. Additionally, a literature review has been conducted. Results: The histological analysis has shown that cryopreservation does not alter the original architecture, and the culture examination that cell viability is successfully preserved. Moreover, both thyroid autotransplantation studies on animals and those on humans that were found in the literature have shown good results regarding the viability and functionality of the transplant. Conclusions: The viability of cryopreserved thyroid tissue found in this study is encouraging. Further studies to evaluate the levels of FT3, FT4 and thyroglobulin in thyroid tissue after cryopreservation are needed to verify that the secretory properties of the thyrocytes have been maintained intact. Furthermore, autotransplanted cases found in the literature do not have a long-term follow-up.

## 1. Introduction

In an effort to provide an alternative solution to lifelong hormone replacement therapy, thyroid autotransplantation studies have been performed both in animals and humans [1]. This therapeutic approach was introduced for the first time in 1938 by Ray [2], although with unsuccessful results. The first successful autotransplantation performed in humans occurred in 1957, thanks to Minuto et al., although the introduction of this procedure into routine clinical practice to date has not yet been achieved [1,3]. Moreover, not all patients are able to stick to the high dosage of levothyroxine of thyroid replacement therapy, which can also have numerous interactions with other medications or with the patient’s diet [4,5,6,7,8,9]. Hence, it is important to avoid over- or undertreatment and meet the body’s needs which may depend on age, residual thyroid function, thyroid stimulating hormone levels, weight, comorbidities, lifestyle and specific events such as pregnancy [7,10,11].

As previously described, cryopreservation is a way of preserving tissues and cells in a state of “suspended viability” while CPAs (cryoprotective agents) such as dimethyl-sulfoxide (DMSO) are used in order to prevent the formation of ice crystals which would damage the structural integrity of the cells [12,13,14].

The idea of autotransplantation was conceived without any association with cryopreservation as an immediate solution for thyroid ectopy [2]. The first study on post-cryopreservation thyroid autotransplantation took place over thirty years later, in 1984, with Pushkar’ et al. [15], followed by a few other studies [16,17].

Immediate or after cryopreservation, heterotopic thyroid autotransplantation could be an alternative solution [1]. This would allow us to use the ability of the thyroid tissue of producing hormones according to the physiological needs of the body [18].

This paper aims to study an alternative solution to lifelong hormone replacement therapy in specific categories of patients who underwent thyroidectomy in childhood or adulthood, with the future perspective of autotransplanting cryopreserved thyroid tissue in selected pediatric patients undergoing thyroidectomy to provide an alternative solution to hormone replacement therapy.

## 2. Materials and Methods

The present paper describes a feasibility study on the effects of the most modern cryopreservation techniques on the structural and functional integrity of follicular cells of thyroid tissue. It was carried out in order to understand whether cell-damaging ice crystals develop and whether organ function is maintained intact.

The present study has been conducted in a single center with collaboration among pediatric surgeons, clinics, pathologists and biologists.

The study was designed taking into account former experience reported in the literature about studies in humans, with particular reference to the work of Shimizu et al. [16] and Kitamura et al. [19]: DMEM (ATCC, Manassas, VA, USA) + 10% FBS (NBS, Sigma Chemical Co., St. Louis, MO, USA) +1 0% DMSO (Merck Co., Rahway, NJ, USA) (where DMEM stands for Dulbecco’s Modified Eagle Medium, DMSO for dimethyl sulfoxide and FBS for fetal bovine serum). In this study, a liquid with 80% BASE, 10% DMSO and 10% human albumin was used. BASE is a support medium for the sample with RPMI 1640 (Gibco BRL Life Technologies, Carlsbad, CA, USA) [20], albumin has a protective function for the cell viability [21] and DMSO has a critical function in preventing the formation of ice crystals during the freezing process [16,19,22,23].

We enrolled in the present study all the patients <18 years of age operated at our institution of hemithyroidectomy or thyroidectomy in a period of 10 months from June 2023. All the patients older than 18 years were excluded. In total, 11 children were enrolled in the study, 9 female and 2 male.

We selected patients undergoing either hemithyroidectomy or thyroidectomy depending on the anatomical extent of the thyroid pathology [24]. For each of these, a fragment of thyroid tissue was taken from the surgical piece for cryopreservation and the surgical piece was sent for histological examination. The sample transport from the operating theater to the Biobank was carried out by storing the material at a controlled temperature of +4 °C, without deviating by more than 1 °C positively or negatively from the optimal transport temperature and always avoiding direct contact between the ice and the sample [25]. The specimen was processed as per the following procedure: BASE128 decontamination (association of vancomycin, gentamicin, cefotaxime and amphotericin B deoxycholate) for 12 h at room temperature. Liquid sample underwent microbiological analysis so as to demonstrate its sterility. At the end of the decontamination, the sample was washed with BASE to remove antibiotic residues.

Lastly, the sample was put into the cryogenic bag together with the freezing liquid containing DMSO, which had also undergone microbiological analysis before being put in contact with the sample. The cryogenic freezing bag was then transferred to a freezer with a programmed temperature reduction (one degree per minute), thus reaching −160 °C in 90 min. The slow freezing technique was chosen because it was identified by Shimizu et al. [16] as the best option for cell recovery. At the end of the freezing procedure, the bags were stored in cryotanks with liquid/gas nitrogen at a temperature between −146 °C and −196 °C. To unfreeze, a bath at 37 °C was prepared so as to immerse the sample and bring it from a temperature of −180 °C to room temperature. Samples for pathological analyses were immersed in formalin, whilst culture analyses were performed in the cell laboratory. The histological study for CASES 8, 9 and 10 and the cultural study for CASE 1 were carried out after the cryopreservation of the samples. CASE 11 underwent a viability test with tetrazolium salts. For the pathological analysis of CASE 1, CASE 8, CASE 9 and CASE 10, the samples were fixed in 10% formalin for 24 h and then completely included in a paraffin block. Histological sections were obtained with a 3 µm thickness, carrying out a histochemical staining with Hematoxylin–Eosin for morphological evaluations under an optical microscope (Nikon, Minako, Tokyo, Japan). For the cultural study, the thyroid samples of CASE 1 and CASE 11 were rinsed with saline, minced with scissors and subjected to the action of collagenases (1 mg/mL, Roche, Basel, Switzerland) for one hour at 37 °C in RPMI 1640 (Whittaker Bioproducts, Inc., Walkersville, MD, USA). The semi-digested follicles were collected, centrifuged and inserted in a six-well plate for culture. Once they reached confluence, they were trypsinized and propagated in 75 cm^2^ flasks with RPMI 1640 medium; fetal bovine serum, FBS, 10% *v*/*v* (Seromed, Biochrom, Cambridge, UK); 2 mM glutamine; and 50 mg/mL penicillin/streptomycin, all positioned in an incubator at 37 °C with 5% CO_2_ [26].

**Figure 1:**
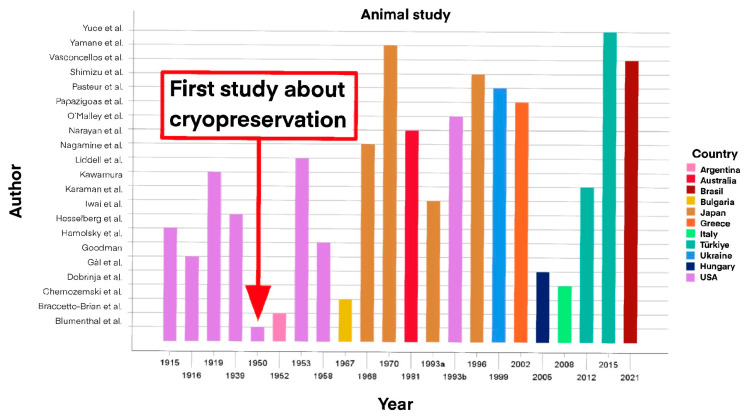
Animal studies divided by year and country of publication [1,9,18,27,28,29,30,31,32,33,34,35,36,37,38,39,40,41,42,43,44,45].

**Figure 2:**
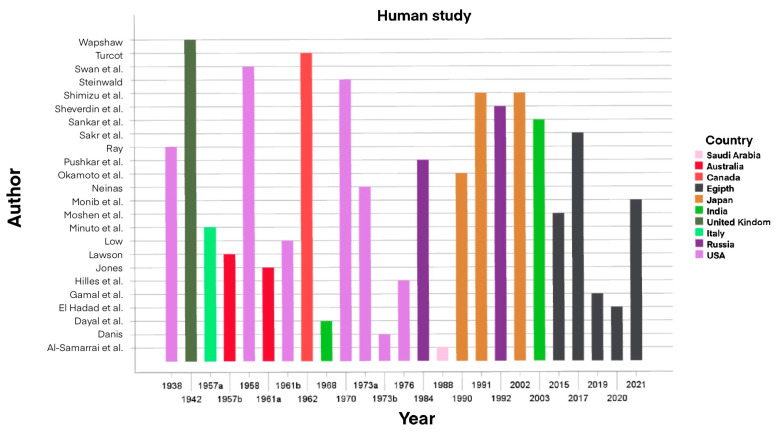
Human studies divided by year and country of publication [1,2,3,15,16,17,46,47,48,49,50,51,52,53,54,55,56,57,58,59,60,61,62,63,64,65,66].

Additionally, a thorough literature review has been conducted on PubMed and EMBASE analyzing studies from 1915 to 2023. We enrolled 22 animal studies and 25 human studies and we divided them by year of publication and by country as shown in Figure 1 and Figure 2.

The studies enrolled have been included in two separate tables reporting them in chronological order, and for each of them the following information were given: authors, year of publication (from 1915 to 2023), nationality of the study, size of the sample, characteristics of the subjects (especially for animals; age, gender and pathology for humans), type of transplant, weight of the transplanted organ (for humans) and outcomes and post-surgical follow-up, one for experimental studies in animals and the other for humans (Table 1 and Table 2).

## 3. Results

Samples were collected from 11 patients (Table 3), nine of which were females and two of which were males, with an average age of 13 years (range 8–18). The following pathologies were observed: three (27%) benign multinodular goiters; seven (64%) nodular goiters, three of which (43%) were papillary carcinomas; and one (9%) MEN2A syndrome with focal C cell hyperplasia. Among the patients with benign thyroid pathology, five (63%) were treated with hemithyroidectomy and three (37%) with thyroidectomy. Malignant thyroid disease has affected three female patients, two (67%) aged 10 years and one (33%) aged 16 years. Two out of three (67%) cases of papillary carcinoma were treated with conservative surgical treatment (lobe-isthmectomy), while the third case (33%) with radical treatment (thyroidectomy). For all of them, histological examination of the surgical specimen and cryopreservation of the thyroid sample were carried out.

For CASE 1, both the morphological and the functional integrity study of the post-cryopreservation sample were performed. For CASES 8, 9 and 10 the morphological integrity study of the post-cryopreservation samples was performed. The post-cryopreservation sample of CASE 11 underwent culture examination. Six samples are still cryopreserved. The samples of CASES 1, 8, 9 and 10 were thawed for histopathological and cultural analyses.

**Figure 3:**
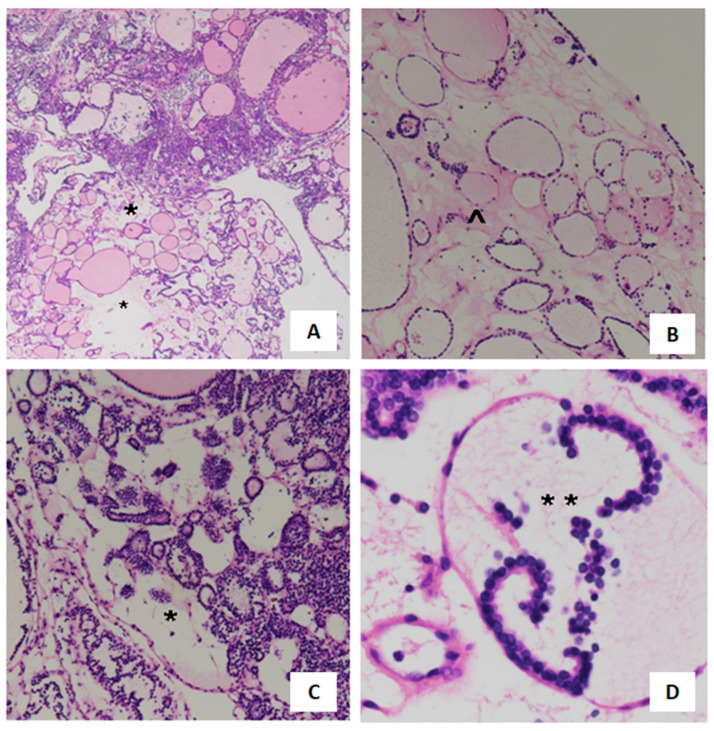
Histological photos of CASE1. Hematoxylin–Eosin staining. Focal regressive micro-macrofollicular aspect (* subfigure (**A**)) focal regressive changes (^ subfigure (**B**)) edema (* subfigure (**C**)) and dislocation of the epithelial cells (* subfigure (**D**)) are shown. Bar (**A**–**C**): 100 µm; (**D**): 20 µm.

The post-cryopreservation pathological analysis of the CASE 1 sample (Figure 3) reports the following: Thyroid tissue with micro-macrofollicular structure and focal regressive aspects (^). Foci of edema affecting the stroma were highlighted (*) in part of the sample, with dissociation and dislocation of the epithelial cells.

**Figure 4:**
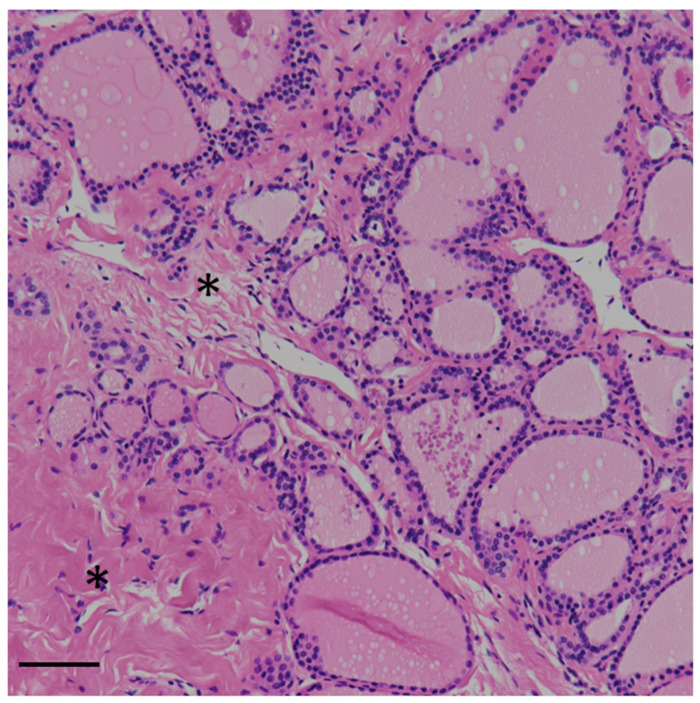
Histological photo of CASE 9 showing minimal and focal edema (*). Hematoxylin–Eosin staining. Bar 50 µm.

The post-cryopreservation pathological analysis of the CASE 9 sample (Figure 4) reports the following: thyroid tissue with micro-macrofollicular histology; no or minimal edema of the stroma; and epithelial cells without significant alterations, i.e., well preserved tissue. (Hematoxylin and Eosin staining, original magnification 4×).

The post-cryopreservation pathological analyses of CASES 8 and 10 report well preserved epithelial cells as well.

**Figure 5:**
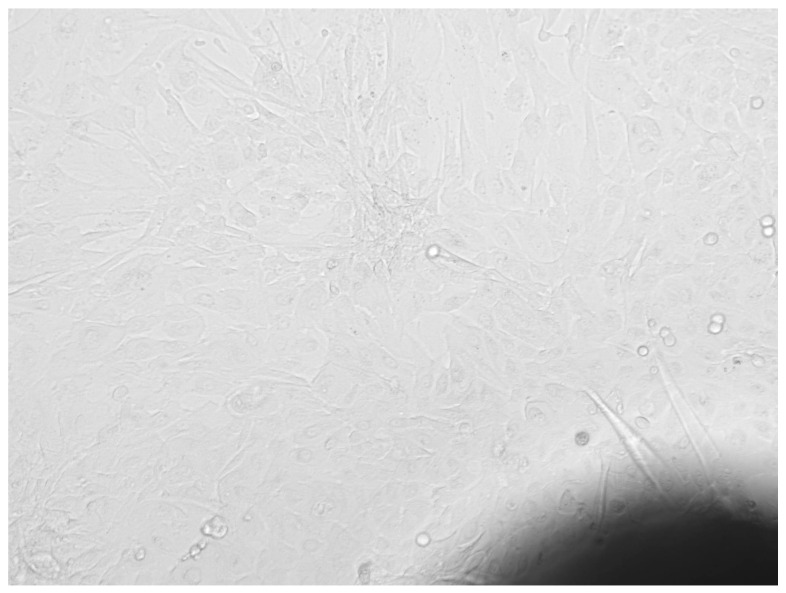
Cell culture photo of CASE 1. The black shadow is the sample. The thyrocytes in the culture grow by expanding from the sample into the medium. Bar 20 µm.

At a preliminary assessment, thyrocytes in the CASE 1 sample under culture are visibly increased in number, consequently considered viable (Figure 5); the proliferation expands from the sample towards the periphery. Thyrocyte behavior in the CASE 11 specimen was deemed similar to the former: cells reaching 80% of confluence were transferred to a medium to perform viability assays in different conditions with tetrazolium salts.

### Literature Review

**Figure 6:**
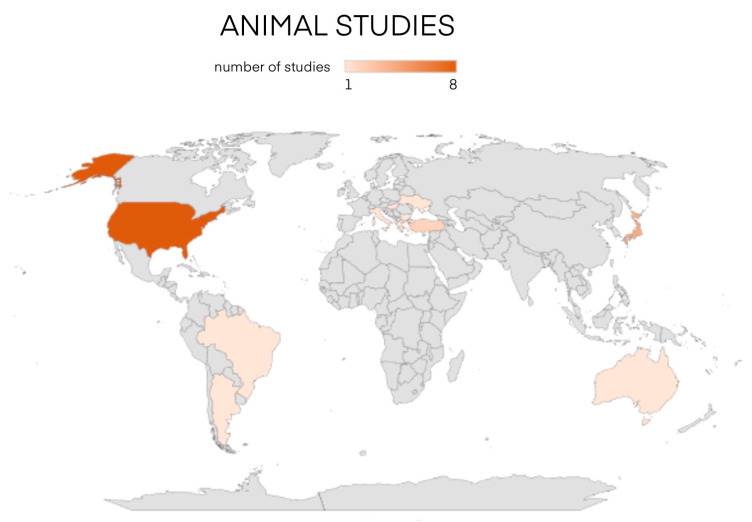
Geographical distribution map of publications on thyroid autotransplantation in animals.

Studies on animals have been collected in Table 1 comprising 22 publications. Studies were clustered according to their geographical distribution, half of which were published by US and European research groups [18,27,28,29,30,31,32,33,34,35,36,37,67] (Figure 6), while the animals enrolled in the studies were mostly murine and canine [28,29,30,31,33,36,37,38,39,40,41,42,67,68], among which only one study discussed orthotopic autotransplantation into the remaining thyroid lobe [33]. In all the other concerned heterotopic autotransplantations, the preferred site was the muscle (posterior limb, neck, and dorsal-abdominal region) [18,32,36,37,38,41,42,43,44,68]. Autotransplantation has been immediate in more than half of the studies [18,27,29,30,31,32,33,34,37,38,39,42,43,44,45,67,68], while post-cryopreservation autotransplantation entailed a minority of cases [27,36,38,41,44,68]. The follow-up of these studies lasted on average two months (range 0.25–12 months) and the animals were monitored at different intervals following surgery, assessing levels of T3, fT3, T4, fT4, TSH, scintigraphy and histological examination.

**Figure 7:**
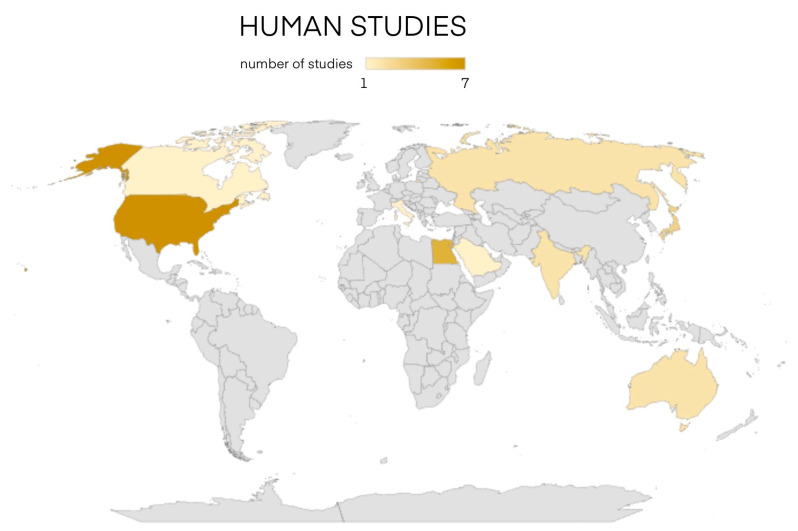
Geographical distribution map of publications on thyroid autotransplantation in humans.

Studies in humans have been collected in Table 2 for a total of 25 publications ranging from 1938 to date [3,69]. Also, these reports were clustered according to their geographical distribution. In Figure 7, it is possible to observe the geographical distribution of the studies, half of which were conducted in the US and Middle East [2,46,47,48,49,50,51,52,53,54,55,56,70]. The sample size for the most numerous study is over 240 patients. The studies entailed patients affected by benign thyroid disease. Specifically, thirteen studies focused on the treatment of lingual ectopy [2,3,46,47,48,49,50,51,57,58,59,60,61,62]; six studies were about patients with multinodular goiter [52,53,54,55,62,70]; six studies were carried out on patients with Graves’ disease [16,17,54,56,63,64,69]; a study on Hashimoto’s thyroiditis [54,69]; two studies on nodular goiter [54,56,69]; a study on unspecified postsurgical hypothyroidism [15] and a study on patients with thyrotoxicosis [65]. The heterograft sites are known in 23 out of 25 cases. The trunk was the most involved site [2,3,47,49,50,51,58,59,62], with prevalence of the rectus abdominalis, followed by the neck [48,50,54,57,60,61,63,64,69], especially the sternocleidomastoid. A less preferred site was the lower limb muscles [52,53,55,56,70], and the upper limb was chosen only once [16]. Immediate autotransplantation was performed in nearly all the studies (22 out of 25—88%) [2,3,47,48,49,50,51,52,53,54,55,56,57,58,59,60,61,62,63,64,65,69], while post-cryopreservation autotransplantation was performed in 3 studies (12%) [15,16,17]. The weight of transplanted thyroid was reported in 10 studies with an average of 7 g (range 0.5–20 g) [16,46,52,53,54,55,56,62,63,64,69]. The engraftment evaluation period following the transplant lasted an average of 6 months (range 5–12). The follow up of these patients had an average duration of 3.5 years (range 1 month–37 years).

## 4. Discussion

Thyroid autotransplantation is a technique that has been studied for over a century and could provide a more physiological alternative to levothyroxine replacement therapy [1].

The oldest studies on immediate autotransplantation were performed on animals in the last years of the nineteenth century [32], while the first study on post-cryopreservation autotransplantation was performed on animals years later, in 1950 [27].

Generally speaking, both thyroid autotransplantation studies on animals and those on humans have shown good results regarding the viability and functionality of the transplant, although information on the outcome remains poor. As the trend of malignant pathology in pediatric age patients is increasing all over the world, an alternative solution to lifelong hormone replacement therapy in specific categories of patients who underwent thyroidectomy could be that of autotransplanting cryopreserved thyroid tissue in selected patients [71,72,73].

The present study was designed taking into account former experience reported in the literature about studies in humans, with particular reference to the work of Shimizu et al. [16] and Kitamura et al. [19] regarding freezing and unfreezing techniques and the two cryopreservation techniques that have been described, such as slow freezing and vitrification, choosing the former [74].

The freezing damage may be more or less extensive on the cell architecture depending on the cell type, freezing speed and unfreezing speed [75]. There are two theories describing cell damage from unprotected freezing [14,76,77,78]. In the first theory, ice crystals are seen as the main cause as they can pierce or separate cells, damaging them through direct mechanical action [76,77], while the second theory concerns osmotic stress in which slow freezing leads to the formation of ice crystals in the extracellular space, with an increase in the concentration of solutes in the liquid fraction causing an osmotic passage of water from the intracellular to the extracellular compartment, leading to the cells’ dehydration [14,78]. The increase in the concentration of intracellular solutes causes the so-called *solute effects injury*, reaching values that are lethal to the cell [14,77,78,79].

Once entered into the cells, the DMSO regulates the water exit, interfering with the hydrogen bonds between individual molecules, thus preventing the ice crystal formation [14,23,79,80].

In 2022, Lee et al. [80] published a study describing the interference of the cryoprotectant on the hydrogen bonds between water molecules: those around DMSO appear disordered, while the hydrogen bonds between distant molecules are partially interrupted. In the case of the thyroid, the cryoprotectant must also be effective in the extracellular space. DMSO, like other cryoprotectants, has the ability to distribute itself throughout the system, with minimal cellular toxicity at low concentrations; however, impaired diffusion into an organ could be the reason for histological alterations [12,14,76,77,81,82].

The material was thawed to be analyzed. Shimizu et al. [16] suggested rapid unfreezing as the best method to reduce the risk of water recrystallization, which would damage the cell viability [17,83]. During the unfreezing process, the concentration of impermeable extracellular solutes decreases, although DMSO increases the cellular content, protecting the cell from excessive volume reduction and from the formation of ice crystals [22,76].

The anatomopathological study was carried out on fragments which measured around 5 mm^3^, an intermediate size between those reported in the literature. In animal studies, Gàl et al. [36] successfully cryopreserved 5 mm^3^ canine thyroid fragments, Yüce et al. [44] preserved fragments of hare thyroid slightly larger than 1 mm^3^ and in rats, Vasconcellos et al. [41] cryopreserved an entire thyroid lobe. Among these, only Vasconcellos et al. [41] and Gal et al. [36] performed a histological examination three and a half and one months after the autotransplant, respectively. In the first case, the histological results with Hematoxylin–Eosin staining and immunohistochemistry with anti-PCNA are available: under the microscope, endocytosis vesicles were visible indicating hormonal activity and muscle tissue between the follicular cells; the positivity at the immunohistochemistry study with anti-PCNA was high [41]. For human studies, we can refer to Kitamura et al. [19] and Shimizu et al. [16]: the first used 1 mm^3^ fragments, while for the second, the histological evaluation of cryopreserved tissue before being transplanted into humans was available and reported a preserved follicular structure. The specimens were adequately thick allowing us to recognize the structure of the thyroid parenchyma; however, the pathological examination of some fragments underwent small focal alterations that had not been priorly highlighted by Shimizu et al. [16]. One explanation could be found in the difference in size of the cryopreserved pieces, which in the aforementioned study can be assumed to be around 1 mm^3^, since they previously collaborated with Kitamura et al. [19].

The cultural analysis of the present study allows us to state that the cells of the sample survived freezing. There is another corroboration of this occurrence in the sample analyzed with the tetrazolium salt which was transformed into formazan in a quantity directly proportional to the cell viability: spectrophotometric analysis revealed that the cells were indeed viable [84,85].

An average quantity of 7 g of tissue should be warranted to perform the autotransplant. This information is important in view of the use in children, in which the thyroid does not have the same size as in adults, but even so, we have to transplant volumes that guarantee the euthyroid state also in adulthood [46]. Since the adult thyroid has an average weight of 16 g (16.4–18.5 in men, 14.4 in women) and an average volume of 14 mL (12–18 mL in men, 10–15 mL in women), one can approximate that the thyroid weighs approximately 1 g/mL [86]. In a 2019 article, reference is made to the 1997 WHO guidelines, which indicated that to obtain an estimate of the correct thyroid volume in children aged 6 to 11 years, it was sufficient to subtract 1 from the age. Therefore, the range 6–11 years would correspond to volume of 5–10 mL; from 12 to 15 years, however, the volume would correspond to the age, thus 12–15 mL [86,87]. Thyroid volumes collected in this study were estimated between 0.8 mL and 2 mL. We managed to remove approximately 0.8–2 g of tissue from the surgical specimen. The aforementioned assumptions lead us to think that it is feasible to collect suitable quantities of thyroid parenchyma, even in a pediatric setting.

Immediate transplantation would have the advantage of being able to be performed in the same session as the thyroidectomy procedure to treat the following benign thyroid pathologies: lingual ectopia of the thyroid, multinodular goiter and Graves’ disease.

**Figure 8:**
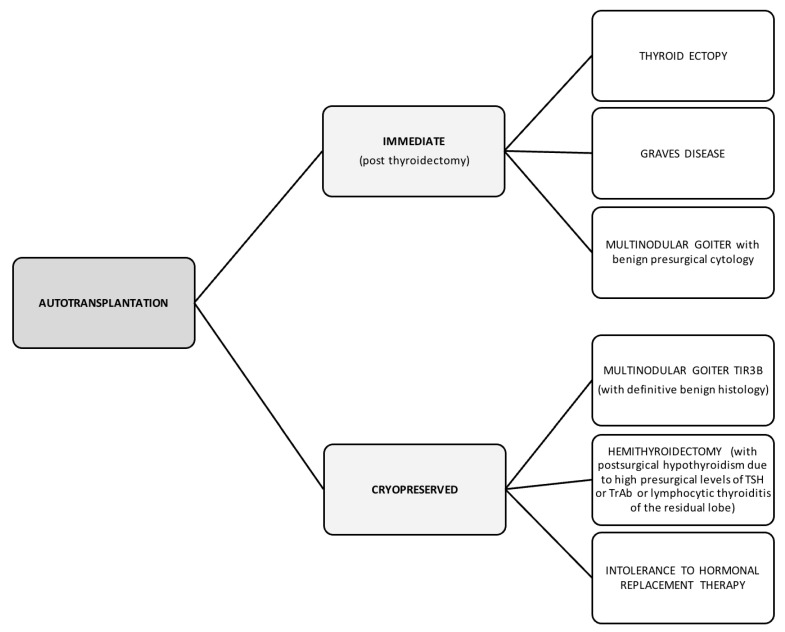
Indications for autotransplantation.

On the other hand, post-cryopreservation transplantation would offer a valid alternative to replacement therapy in cases of patients undergoing thyroidectomy in which it is necessary to exclude a diagnosis of malignancy [44]. This method could then be extended to patients with TIR3B lesions which on post-operative histology result to be benign [88]. A previous study reported success after cryopreservation autotransplantation in a case suffering from Graves’ disease: not only optimal organ function was maintained, but the patient’s anti-thyroid antibody concentrations were also reduced to a stable level slightly higher than normal [16]. Hemithyroidectomy cases that are at high risk of developing hypothyroidism after surgery, in which thyroiditis with lymphocytic infiltration in the residual lobe and/or elevated pre-surgical TSH levels and/or elevated anti-thyroid antibody levels are found [89,90,91], could benefit from autotransplantation after cryopreservation. Another group might be represented by the patients who are hypersensitive to the active or inactive components of hormone replacement treatment [92,93,94,95]. The candidates for the immediate autotransplantation or post-cryopreservation techniques are summarized in Figure 8.

The first study on animals of immediate thyroid autotransplantation was performed in 1915 on guinea pigs by Hesselberg, although the author reports that the idea of thyroid autotransplantation had already arisen at the end of the 1800s. Hesselberg studied the engraftment of thyroid tissue after autotransplantation or allotransplantation: after 52 days of observation, the allografts had disappeared, while the autografts were trophic [32]. Another thyroid autotransplantation study on guinea pigs with thyroidectomy was conducted by Karaman et al. in 2012 without signs of post-surgical hypothyroidism: one month after the operation, he recorded low levels of thyroid hormone and high levels of TSH, while towards the eighth week, the values reversed with low TSH levels and normal thyroid hormone levels, with functional recovery of the organ [43].

Canine studies of thyroid transplantation preserving the vascular pedicle and making subsequent anastomosis were carried out by Goodman in 1916 and by Kawamura in 1919 [30,31]. In 1968, Nagamine et al. returned to focus on vascular microanastomoses to transplant the tissue without having to fragment it, indicating vessels of 3 mm in external diameter as adequate to support the tissue: from the histological study of the autotransplants, signs of suffering emerged in the first days immediately following the operation, with recovery in the following two weeks, accompanied by functional recovery, although the animal model enrolled in this study is not known [42]. O’Malley et al. performed a direct orthotopic autotransplantation in the residual thyroid lobe on dogs in 1993 using isolated thyroid follicles with positive results 14 days later. This was the only study about orthotopic transplantation [33].

In 1939, Ingle et al. sutured the thyroid to the ovary of adult rats after total or partial thyroidectomy, highlighting a hyperplasia of the transplanted tissue [67]. Other studies on immediate autotransplant in rats were performed in 1952 by Hamolsky et al. and in 1993 by Iwai et al. [29,39].

Chernozemski et al. [34] in 1967 studied thyroid follicles in autografts in hamsters, noting that they were smaller than those in allografts.

In 1981, Narayan et al. [45] performed an in vivo report in which he observed the morphology and ultrastructure of the thyroid autograft in rabbits, whereas in 2002, Papaziogas et al. highlighted a period of decrease in thyroid activity immediately following the autotransplant, with functional recovery after four weeks, interpreting it as the necessary time for the development of the revascularization of the autograft [18].

In 1950, Blumenthal et al. made the first study on cryopreservation, highlighting how cryopreservation in nitrogen at −190 °C was better than that at −70 °C. The authors also demonstrated that an organ could maintain its vital functions intact after freezing, despite the complication of ice crystals; they administered TSH before/after the autotransplant, to promote survival [27]. In 1996, Shimizu et al. performed the second study on post-cryopreservation autotransplantation, in which he recorded a functional recovery of the organ two months after the operation [1,38]. In 2005, Gal et al. performed the post-cryopreservation transplant on dogs, confirming the presence of a period of decrease in thyroid activity, followed by a functional recovery of the organ (in this case after a month): six days after the autotransplant, the thyroid hormone levels had almost dropped to zero, rising again after eight days and returning to pre-operative levels a month later [36]. In 2015, Yüce et al. [44] repeated the experiment on rabbits, performing immediate autotransplantation in one group and cryopreserved autotransplantation in another: while in the first group he obtained five out of six viable transplants, in the second group he managed to demonstrate viability only in a case; furthermore, the rabbits in the first group returned to being euthyroid after 8 months, unlike the rabbits in the second group, despite the increasing trend in hormonal levels. In 2021, Vasconcellos et al. performed a study on post-cryopreservation thyroid autotransplantation in rats, because they have similar thyroid anatomy, histology and physiology to those of humans, suggesting to investigate the relationship between the duration of cryopreservation and the results of the autograft [41]. In 2022, Schanaider et al. performed a successful study on post-cryopreservation thyroid autotransplantation in rats using Scintigraphy with 99mTcO4 to evaluate the functionality of thyroid grafts [68].

In 1999, Pasteur [35] performed an in vitro assay in which he demonstrated the maintenance of the morphological and functional integrity of the pig thyroid in culture, although it is not clear whether the analyzed tissue followed an immediate graft or cryopreservation.

The first documented study on immediate thyroid autotransplantation in humans was that of 1938 by Ray, although the lingual ectopy of the thyroid was initially mistaken for a tumor and treated with radiotherapy; in the next attempt the autotransplant was made into the rectus abdominis muscle but failed—there was an infection and tissue extrusion [2]. In 1942, Wapshaw attempted immediate thyroid autotransplantation in lingual ectopy again: in the first seven months, the patient showed signs of initial myxedema, but over a year after the surgery the picture frankly shifted towards hypothyroidism [61].

The first successful study performed in humans dates back to 1957, with Minuto et al. in Italy, on an eighteen-year-old patient with lingual ectopia of the thyroid. The autotransplant was performed at the level of the rectus abdominis muscle and after five months, the patient returned to being euthyroid and maintained that state also at the follow up visits over the following 37 years [3]. This technique for the treatment of lingual ectopy was then applied in many other studies [46,47,48,49,50,51,57,58,59,62,70].

Among these, there is the first pediatric case, a 7-year-old girl, reported in two articles by Swan: the heterotopic autograft was initially successful, but after a few years the girl showed clear radiographic signs of delayed bone growth, perhaps due to insufficient quantity of parenchyma and to the anatomical site not being very favorable for neoangiogenesis [46,47]. In 1957, Lawson reported another case of transplant in an 11-year-old girl, with a positive outcome even after nine months, but there were no further updates [51]. In 1961, Jones performed the autotransplant on a 9-year-old girl and the graft was found to be viable five weeks after surgery; after two years, the patient was in full pubertal development, although menarche had not yet appeared [58]. In the same year, Low performed the autotransplant on a 2-year-old infant: six months after surgery, the transplant picked up radioiodine and a year later the patient was healthy [48]. In 1962, Turcot performed the autotransplant on two pediatric patients, recording radioiodine uptake two months later, but insufficient thyroid activity, for which he resorted to replacement therapy [59]. In 1968, Dayal et al. positioned the autograft in the capsule of the submandibular gland and in the neck of a 20-year-old patient, with positive results one year later [57]. In 1970, Steinwald et al. reported a 9-year-old patient to be already clinically euthyroid 3 months after the operation; at 7 months, a biopsy was performed, and the histology confirmed the secretory activity of the transplant [49]. In 1973, Danis [66] reported the case of an 8-year-old child with lingual ectopia, autotransplanted at the level of the thigh, who remained euthyroid for a documented period of seven years. In the same year, Neinas performed the autotransplantation in two pediatric patients: one failed, while the other was accompanied by the administration of TSH for the first nine postoperative days; the positive result suggested that TSH facilitated the engraftment of the autograft [50]. In 2005, Al-Samarrai et al. [60] performed immediate autotransplantation on a 9-year-old girl, who reached the euthyroid state in 4 months.

In 1976, Hilles et al. [62] successfully operated on a 39-year-old woman with ectopic multinodular goiter: when she was checked eight months later, the woman appeared euthyroid. Also, other more recent works studied autotransplant in patients with multinodular goiter. In 2017, Mohsen et al. [55] performed autotransplants in 40 patients, 12 of whom received 5 g of tissue, while the others, 10 g. The patients were then subjected to replacement therapy, suspended 3 months before each check-up (2, 6 and 12 months). The 10 g group showed greater Tc-99m uptake, while there were no statistically significant differences in TSH levels. However, FT3 levels proved to be higher in the 10 g group after 12 months; FT4 levels were also higher in the 10 g group, although not in a statistically significant manner [55]. After one year, three patients with the 5 g transplant and five patients with the 10 g transplant reached close-to-normal TSH and suspended replacement therapy, entering a follow-up with checks every three months [55]. In 2020, El Hadad et al. performed 5 g and 10 g autotransplants in 40 patients. One year after the operation, 41% of patients of the first group and 88% of the second group were euthyroid [53]. In 2021, Monib et al. administered levothyroxine after surgery until the thyroid function was stabilized: one year after the operation, patients under 50 were found to be euthyroid, while those over 50 needed a small dose of levothyroxine; furthermore, patients who had received a greater quantity of thyroid parenchyma had reached the euthyroid state in a shorter time [52].

In 1990, Okamoto et al. [64] performed the first autotransplant at the same time as the subtotal thyroidectomy in five patients with Graves’ disease: it was not possible to document scintigraphic uptake by the transplant only for one of them [64].

In 2003, Sankar et al. transplanted patients with multinodular goiter or Graves’ disease: all patients with GMN were euthyroid after 6 months, regardless of whether the transplant showed uptake (six out of eight) or not (two out of eight) on scintigraphy. On the contrary, six out of seven patients with Graves’ disease were hypothyroid after 6 months, perhaps due to the insufficient size of the transplant or to lymphocytic infiltration [63].

In 1992, Sheverdin et al. [65] carried out the largest study we have, with 246 patients (adults and children) with thyrotoxicosis, to prevent postoperative hypothyroidism: the symptoms of thyrotoxicosis resolved in all patients and 3.2% of patients showed a decrease in thyroid activity in the first six months after the operation.

Other recent studies were performed on patients with non-specified benign disorders. Sakr et al. [1] cited Saleh’s dissertation [96], which is believed to have been updated in the 2018 article: Sakr et al. [56] described the immediate autograft in 20 patients, 2 of whom received a diagnosis of papillary carcinoma after the operation and subsequent removal of the autograft; after a year, 5 patients had to resort to replacement therapy. In 2019, Gamal et al. performed an immediate autotransplantation in 30 patients. Six to nine months after the operation, 15 patients were evaluated with Tc-99m. Of these, 13 were found to be functioning, while 2 were not; 6–9 months after the transplant, 27 patients were found to be euthyroid, while 3 were hypothyroid. It is believed that the work of Kotb et al. published in 2022 is an update of the study by Gamal et al. [54,69].

In 1984, Pushkar’ et al. performed the first study on post-cryopreservation thyroid autotransplantation, focusing on post-surgical hypothyroidism. The thyroid samples were cryopreserved for a period of 4–12 months. [15] In 1991, Shimizu et al. performed a post-cryopreservation autotransplantation in a patient with Graves’ disease [17]. In 2002, Shimizu et al. published a study with four patients, in which he urged us to reflect on the fact that in the cases treated with subtotal thyroidectomy, the euthyroid state was not exclusively the result of the autotransplant but also of the thyroid tissue remaining in situ [16].

The results of the studies found in the literature show that autotransplantation is a technique with high potential, although it is necessary to deepen our knowledge and improve the technique; furthermore, the follow-ups of most of these patients are often too short to have a prospective evaluation.

The main limitation of our study is the small number of patients and the fact that the functional properties of the autotransplanted tissues had not been proved yet. The patient sample has to be extended in future studies in order to reach more reliable results.

Further studies need also to evaluate the levels of FT3, FT4 and thyroglobulin in thyroid tissue after cryopreservation and might verify that the secretory properties of the thyrocytes have been maintained intact. What is expected is in fact that the thyroid hormones will be found free in the culture supernatant, as the thyroid hormone transporters are not available in this extracorporeal system.

## 5. Conclusions

Patients who could benefit from immediate autotransplantation could be those who have undergone thyroidectomy for benign pathologies. Patients who could benefit from post-cryopreservation transplantation could be those in whom a diagnosis of malignancy was excluded during the postoperative histological examination despite an initial suspicious cytological examination (TIR3B), patients at high risk of post-surgical hypothyroidism and patients intolerant to hormone replacement treatment.

To date, the results obtained with this study are encouraging, as the histological analysis has shown that it does not distort the original architecture, and the culture examination has highlighted cell viability.

Further studies are needed to prove the functional capacity of the transplanted tissue and to extend the sample of patients in the exam in order to obtain more solid results.

## Figures and Tables

**Table 1:** Studies in animals.

Authors	Year	Country	Animal	No. of Animals	Site of Transplant	Surgery	Follow-Up (Months)	Success
Hesselberg et al. [32]	1915	USA	Guinea pigs	75	Abdomen	Immediate autotransplant Immediate allotransplant	0–2	Good
Goodman [31]	1916	USA	Dogs	3	Vessels	Immediate autotransplant	1<	Dead
Kawamura [30]	1919	USA	Dogs	8 7	Vessels	Immediate autotransplant Immediate allotransplant	2	Dead
Ingle et al. [67]	1939	USA	Rats	22	Ovary	Immediate autotransplant	0–3	Good
Blumenthal et al. [27]	1950	USA	Guinea pigs	12 12	Subcutis	Post-cryopreservation Autotransplant at −70 °C Post-cryopreservation Autotransplant at −190 °C Immediate autotransplant	0.5	1 8 <1
Brachetto-Brian et al. [40]	1952	Argentina	Rats	-	Spleen	Non-specified autotransplant	-	-
Liddle et al. [28]	1953	USA	Dogs	-	-	Non-specified autotransplant	-	-
Hamolsky et al. [29]	1958	USA	Rats	-	Armpit Spleen	Only thyroidectomy Immediate autotransplant Sham surgery	Up to 12	Good
Chernozemski et al. [34]	1967	Bulgaria	Syrian hamsters	13 16 19	Cheek	Immediate autotransplant Allotransplant Allotransplant (without thyroidectomy)	1.5–3	12 13 4
Nagamine et al. [42]	1968	Japan	Dogs	100	Bikini lineNeck	Immediate autotransplantSham surgeryThyroid denervationLymphatics binding	>2	Yes
Narayan et al. [45]	1981	Australia	Rabbits	17	Ear	Immediate autotransplant	11	Good
Iwai et al. [39]	1993	Japan	Rats	8	Spleen and retroperitoneal fat	Immediate autotransplant	0.7	Positive
O’Malley et al. [33]	1993	USA	Dogs	-	Thyroid	Immediate orthotopic autotransplant	0.5	Positive
Shimizu et al. [38]	1996	Japan	Rats	-	Renal capsule or muscles	Immediate autotransplant Cryopreserved autotransplant	1.5–2	Both
Pasteur et al. [35]	1999	Ukraine	Pig	-	In vitro	-	1	Yes
Papaziogas et al. [18]	2002	Greece	Rabbits	10 10 10 8	Quadriceps Rectus abdominis Back muscle	Immediate autotransplant Control	2	Very good
Gál et al. [36]	2005	Hungary	Dogs	12	Sternocleidomastoid and greater omentum	Cryopreserved autotransplant	1	Yes
Dobrinja et al. [37]	2008	Italy	Rats	60	Rectus abdominis	Immediate autotransplant Non-specified autotransplant	1	70%
Karaman et al. [43]	2012	Turkey	Guinea pigs	6 6 6 6	Back muscle	Incision only Thyroidectomy Immediate autotransplant Heterotransplant	2	Yes
Yüce et al. [44]	2015	Turkey	Rabbits	16	Quadriceps	Immediate autotransplant Cryopreserved autotransplant	2	Euthyroid AI (autotrap) Increasing AC (cryopreserved autotransplant)
Vasconcellos et al. [41]	2021	Brazil	Male albino rats	8 8 8 8	Biceps femoris muscle	Control Sham surgery Thyroidectomy Cryopreserved autotransplant	3.3	8
Schanaider et al. [68]	2022	Brazil	Rats	8 8 8	Biceps femoris muscle	Control Thyroidectomy Cryopreserved autotransplant	3.5	Yes

**Table 2:** Studies in humans.

Author	Year	Country	No. of Patients	Patient’s Age (Years)	Sex	Pathology	Transplant Site	Surgery	Weight (g)	Months to Return to Euthyroid	Follow-Up Length (Months)
Ray [2]	1938	USA	1	39	M	Lingual thyroid ectopia	Rectus abdominis	Immediate autotransplant	-	Failure	-
Wapshaw [61]	1942	England	1	20	F	Lingual thyroid ectopia	Neck	Immediate autotransplant	-	Failure	>12
Minuto et al. [3]	1995	Italy	1	18	F	Lingual thyroid ectopia	Rectus abdominis	Immediate autotransplant	-	5 months	444
Lawson [51]	1957	Australia	2	11	F	Lingual thyroid ectopia	Rectus abdominis	Immediate autotransplant	-	-	9
Swan et al. [46,47]	1958	USA	1	7	F	Lingual thyroid ectopia	Rectus abdominis	Immediate autotransplant	2	6 months	96
Jones [58]	1961	Australia	1	9	F	Lingual thyroid ectopia	Rectus abdominis	Immediate autotransplant	-	1	24
Low [48]	1961	USA	1	2	F	Lingual thyroid ectopia	Sternocleidomastoid	Immediate autotransplant	-	6	6
Turcot [59]	1962	Canada	2	514	F	Lingual thyroid ectopia	Rectus abdominis	Immediate autotransplant	-	Transplant failure	2
Dayal et al. [57]	1968	India	1	20	F	Lingual thyroid ectopia	Submandibular glands neck	Immediate autotransplant	-	-	12
Steinwald [49]	1970	USA	1	9	F	Lingual thyroid ectopia	Pectoralis major and rectus abdominis	Immediate autotransplant	-	-	7
Neinas [50]	1973	USA	2	-	F	Lingual thyroid ectopia	Neck and abdomen	Immediate autotransplant	-	1 failure -	1
Danis [66]	1973	USA	1	8	M	Lingual thyroid ectopia	Thigh	Immediate autotransplant	-	-	72
Hilles et al. [62]	1976	USA	1	39	F	Lingual thyroid ectopia with nodular goiter	Rectus abdominis	Immediate autotransplant	20	-	8
Pushkar’ et al. [15]	1984	Russia	-	-	-	Post-surgical hypothyroidism	-	Post-cryopreservation (4–12 months) autotransplant	-	-	18
Al-Samarrai et al. [60]	1988	Saudi Arabia	1	9	F	Lingual thyroid ectopia	Neck	Immediate autotransplant	-	4	5,5
Okamoto et al. [64]	1990	Japan	5	39 34 28 26 26	2M 3F	Graves’ disease	Sternocleidomastoid or neck muscles	Immediate autotransplant (with STT)	0.5–2	-	26.4–84
Shimizu et al. [17]	1991	Japan	1	-	-	Graves’ disease	-	Post-cryopreservation autotransplant post-STT	-	-	-
Sheverdin et al. [65]	1992	Russia	246	Children and adults	-	Thyrotoxicosis	-	Immediate autotransplant vs. Thyroidectomy	-	3,2% of patients, 6 months in transplants vs. 6,6% non-treated patients	2–180
Shimizu et al. [16]	2002	Japan	4	58 45 21 34	3F 1M	Graves’ disease	Forearm	Post-cryopreservation autotransplant post-STT	2.5–3.5	6 (3 out of 4 patients)	-
Sankar et al. [63]	2003	India	15	-	-	7 Graves’ disease 8 Multinodular goiter	Sternocleidomastoid	Immediate autotransplant with STT	3–5	6 months in functioning transplanted patients (9 out of 15)	6
Mohsen et al. [55]	2017	Egypt	40	-	-	Multinodular goiter	Thigh	Immediate autotransplant	5–10	Some months	12
Sakr et al. [1,56]	2017	Egypt	20	24–49	15 F 5 M	13 Multinodular goiter 4 Graves’ disease 3 Nodular goiter	Thigh	Immediate autotransplant	10–15	2–12 (2 papillary carcinomas with transplant removal)	12
Gamal et al. [54] Mohamed Kotb et al. [69]	2019 2022	Egypt	30	20–55	7 M 23 F	4 Graves’ disease 2 Hashimoto 24 Nodular goiter	Sternocleidomastoid	Immediate autotransplant	2–5	6	12
El Hadad et al. [53]	2020	Egypt	40	-	-	Multinodular goiter	Thigh	Immediate autotransplant	5–10	2–12	12
Monib et al. [52]	2021	Egypt	40	24–53	F	Multinodular goiter	Thigh	Immediate autotransplant	10–15	2–12	12

**Table 3:** Table of cases recruited for the study.

CASES	Gender	Age (Years)	Thyroid Pathology	Cytological Examination	Surgery	Histological Examination
CASE1	F	13	Multinodular goiter of the left lobe	TIR2A nodule	Left hemithyroidectomy	Micro-macrofollicular nodule with hyperplastic aspects and collateral multinodular thyroid parenchyma
CASE2	F	16	Nodular goiter of the left lobe	TIR2 oxyphilic cell nodule	Left lobe-isthmectomy	Papillary carcinoma (3 cm) follicular variant with solid aspects (30%), capsulated, multifocal
CASE3	F	17	Nodular goiter of the right lobe	TIR3B oxyphilic cell microfollicular nodule	Right lobectomy	Micro-macrofollicular thyroid parenchyma
CASE4	F	13	Isthmic nodular goiter of the right lobe	TIR3A isthmic thyroid nodule with microfollicular cytology	Right lobe-isthmectomy	Micro-macrofollicular isthmic nodule with marked hyperplastic aspects and regressive phenomena
CASE5	F	9	Nodular goiter of the right lobe	Nodule in the right lobe of the thyroid TIR2	Right lobectomy	Micro-macrofollicular nodule with widespread and marked hyperplastic aspects and focal regressive phenomena
CASE6	F	10	Nodular goiter of the left lobe	Papillary thyroid carcinoma TIR5	Left lobe-isthmectomy	Classic variant papillary carcinoma focally infiltrating the loose perithyroid tissues and, microscopically, the muscular tissues; multifocal; presence of widespread lymphatic and vascular embolization (No. emboli < 4) and multiple lymph node metastases
CASE7	M	15	Bilateral multinodular goiter	TIR3A thyroid nodule	Near total thyroidectomy	Bilateral micro-macrofollicular nodules with marked hyperplastic aspects; collateral multinodular thyroid parenchyma
CASE8	M	8	MEN2A	-	Total thyroidectomy	Focal C cell hyperplasia
CASE9	F	10	Multinodular goiter of the right lobe	TIR3B microfollicular architecture nodule	Total thyroidectomy	Classic variant papillary carcinoma (1.5 cm), capsulated, bilateral in multinodular goiter
CASE10	F	18	Nodular goiter of the right lobe	TIR3B cytology nodule	Right lobectomy	Microfollicular adenoma
CASE 11	F	12	Nodular goiter of the right lobe	TIR3 cytology nodule	Thyroidectomy	Microfollicular adenoma with hyperplastic aspects

## Data Availability

The data presented in this study are available in this article.
